# Einheitliche Basisversorgung von Kindern und Jugendlichen mit *Long COVID*: Stellungnahme einer multidisziplinären Arbeitsgruppe der DGKJ-Konvent-Gesellschaften (Stand: Februar 2022)

**DOI:** 10.1007/s00112-021-01408-1

**Published:** 2022-05-25

**Authors:** Nicole Töpfner, Deutsche Gesellschaft für Pädiatrische Infektiologie e. V. (DGPI), Martin Alberer, Tobias Ankermann, Gesellschaft für Pädiatrische Pneumologie e. V. (GPP), Stephan Bender, Deutsche Gesellschaft für Kinder- und Jugendpsychiatrie, Psychosomatik und Psychotherapie e. V. (DGKJP), Reinhard Berner, Jan de Laffolie, Gesellschaft für Pädiatrische Gastroenterologie und Ernährung e. V. (GPGE), Jens Dingemann, Deutsche Gesellschaft für Kinderchirurgie e. V. (DGKCH), Dirk Heinicke, Bündnis Kinder- und Jugendreha e. V. (BKJR), Johannes Peter Haas, Gesellschaft für Kinder- und Jugendrheumatologie (GKJR), Markus Hufnagel, Thomas Hummel, Deutsche Gesellschaft für Hals-Nasen-Ohren-Heilkunde, Kopf- und Hals-Chirurgie e. V. (DGHNO-KHC), Hans-Iko Huppertz, Deutsche Akademie für Kinder- und Jugendmedizin (DAKJ), Markus Knuf, Robin Kobbe, Thomas Lücke, Gesellschaft für Neuropädiatrie e. V. (GNP), Joachim Riedel, Deutsche Gesellschaft für Sozialpädiatrie und Jugendmedizin (DGSPJ), Josef Rosenecker, Deutsche Gesellschaft für Pädiatrische Rehabilitation und Prävention e. V. (DGpRP), Joachim Wölfle, Deutsche Gesellschaft für Kinderendokrinologie und -diabetologie e. V. (DGKED), Barbara Schneider, Deutsche Gesellschaft für Schlafforschung und Schlafmedizin e. V. (DGSM), Dominik Schneider, Deutsche Gesellschaft für Kinder- und Jugendmedizin e. V. (DGKJ), Valentin Schriever, Deutsche Gesellschaft für Hals-Nasen-Ohren-Heilkunde, Kopf- und Hals-Chirurgie e. V. (DGHNO-KHC), Anne Schroeder, Gesellschaft für Neuropsychologie (GNP), Silvia Stojanov, Tobias Tenenbaum, Stefan Trapp, Berufsverband der Kinder- und Jugendärzte e. V. (BVKJ), Daniel Vilser, Deutsche Gesellschaft für Pädiatrische Kardiologie und Angeborene Herzfehler e. V. (DGPK), Folke Brinkmann, Uta Behrends

**Affiliations:** 1 Klinik und Poliklinik für Kinder- und Jugendmedizin, Klinikum Rechts der Isar (AöR) der Technischen Universität München und München Klinik gGmbH, München, Deutschland; 2 Klinik für Kinder- und Jugendmedizin, Katholisches Klinikum Bochum gGmbH, Standort St. Josef-Hospital, Alexandrinenstr. 5, 44791 Bochum, Deutschland

**Keywords:** SARS-CoV-2, Long COVID, Kinder, Konsensus, Deutschland, SARS-CoV-2, Long COVID, Children, Consensus, Germany

## Abstract

Das vorliegende Konsensuspapier bietet in Ergänzung zur AWMF-S1-Leitlinie eine Übersicht über die verschiedenen klinischen Aspekte von *Long COVID* im Kindes- und Jugendalter. Es wurde von Vertreter:innen aus 19 Fachgesellschaften des DGKJ-Konvents und kooperierenden Fachgesellschaften erstellt und bietet Expertenempfehlungen für die Praxis auf Grundlage der bisherigen, noch geringen studienbasierten Evidenz zu *Long COVID* im Kindes- und Jugendalter. Es enthält Screeningfragen zu *Long COVID* sowie einen Vorschlag zur strukturierten, standardisierten pädiatrischen Anamnese und zur diagnostischen Evaluation bei V. a. *Long COVID*. Dazu werden ein zeit- und ressourcensparender Erfassungsbogen, der die Komplexität des Krankheitsbildes berücksichtigt, über die Internetseiten der DGKJ und DGPI zur Verfügung gestellt und weitere Fragebögen zur Abklärung von spezifischen neurokognitiven und/oder psychischen Störungen sowie post-exertioneller Malaise (PEM) und myalgischer Enzephalomyelitis/chronischem Fatigue-Syndrom (ME/CFS) benannt. Anhand der jeweiligen anamnestisch und klinisch ermittelten Hauptsymptome werden ein gestuftes, diagnostisches Vorgehen und eine multidisziplinäre Betreuung empfohlen.

**Zusatzmaterial online:**

Die Online-Version dieses Beitrags (10.1007/s00112-021-01408-1) enthält weitere Tabellen mit Angaben zur erweiterten Diagnostik (Labor- und Funktionsdiagnostik, Bildgebung).

## Abstract (en)

This current consensus paper for long COVID complements the existing AWMF S1 guidelines for long COVID with a detailed overview on the various clinical aspects of long COVID in children and adolescents. Members of 19 different pediatric societies of the DGKJ convent and collaborating societies together provide expert-based recommendations for the clinical management of long COVID based on the currently available but limited academic evidence for long COVID in children and adolescents. It contains screening questions for long COVID and suggestions for a structured, standardized pediatric medical history and diagnostic evaluation for patients with suspected long COVID. A time and resource-saving questionnaire, which takes the clinical complexity of long COVID into account, is offered via the DGKJ and DGPI websites as well as additional questionnaires suggested for an advanced screening of specific neurocognitive and/or psychiatric symptoms including post-exertional malaise (PEM) and myalgic encephalomyelitis/chronic fatigue syndrome (ME/CFS). According to the individual medical history as well as clinical signs and symptoms a step by step diagnostic procedure and a multidisciplinary therapeutic approach are recommended.

## Infobox 1 Information: Beteiligte Fachgesellschaften

- Deutsche Gesellschaft für Pädiatrische Infektiologie e. V. (DGPI)
- Deutsche Gesellschaft für Kinder- und Jugendmedizin e. V. (DGKJ)
- Gesellschaft für Pädiatrische Pneumologie e. V. (GPP)
- Deutsche Gesellschaft für Kinder- und Jugendpsychiatrie, Psychosomatik und Psychotherapie e. V. (DGKJP)
- Gesellschaft für Pädiatrische Gastroenterologie und Ernährung e. V. (GPGE)
- Deutsche Gesellschaft für Kinderchirurgie e. V. (DGKCH)
- Bündnis Kinder- und Jugendreha e. V. (BKJR)
- Gesellschaft für Kinder- und Jugendrheumatologie (GKJR)
- Deutsche Gesellschaft für Hals-Nasen-Ohren-Heilkunde, Kopf- und Hals-Chirurgie e. V. (DGHNO-KHC)
- Deutsche Akademie für Kinder- und Jugendmedizin (DAKJ)
- Arbeitsgemeinschaft Pädiatrische Immunologie e. V. (API)
- Gesellschaft für Neuropädiatrie e. V. (GNP)
- Deutsche Gesellschaft für Sozialpädiatrie und Jugendmedizin (DGSPJ)
- Deutsche Gesellschaft für Pädiatrische Rehabilitation und Prävention e. V. (DGpRP)
- Deutsche Gesellschaft für Kinderendokrinologie und -diabetologie e. V. (DGKED)
- Deutsche Gesellschaft für Schlafforschung und Schlafmedizin e. V. (DGSM)
- Gesellschaft für Neuropsychologie (GNP)
- Berufsverband der Kinder- und Jugendärzte e. V. (BVKJ)
- Deutsche Gesellschaft für Pädiatrische Kardiologie und Angeborene Herzfehler e. V. (DGPK)

## Einführung

Ende Januar 2020 wurde das *„severe acute respiratory syndrome coronavirus type 2“* (*SARS-CoV‑2*) zum ersten Mal in Deutschland nachgewiesen. Bis Ende Februar 2022 haben sich nach Daten des Robert Koch-Instituts (RKI) über 14 Mio. Menschen in Deutschland mit diesem Virus infiziert, darunter mehr als 3,5 Mio. Kinder und Jugendliche [15]. Die Infektion kann in jedem Lebensalter asymptomatisch verlaufen oder eine *„coronavirus disease 2019“* (*COVID-19*) verursachen. Im Vergleich zu Erwachsenen im fortgeschrittenen Alter oder Patient*innen mit bestimmten Grunderkrankungen kommt es bei Kindern und Jugendlichen selten zu schwerer *COVID-19*. Die Raten für Hospitalisierung (< 2 %) und Mortalität (< 0,03 %) sind bisher gering [15]. In einem Survey der Deutschen Gesellschaft für Pädiatrische Infektiologie (DGPI) wurden seit Beginn der Pandemie in Deutschland von den 180 teilnehmenden Zentren bis KW 8/2022 über 4000 hospitalisierte pädiatrische Fälle von COVID-19 mit detaillierten klinischen Informationen erfasst [11]. Von diesen benötigten 4 % eine intensivmedizinische Behandlung. Das RKI listet bis KW 7/2022 12.801 stationär behandelte Kinder im Alter von 0 bis 14 Jahren [15]. Eine seltene Besonderheit, die vor allem für das Kindesalter berichtet wird, ist das Auftreten eines *„multisystem inflammatory syndrome in children“* (*MIS‑C*, Synonym: *„pediatric inflammatory multisystem syndrome temporally associated with SARS-CoV-2“*, kurz *PIMS-TS*)(ICD-10 U10.9), welches meist zwischen 2 und 6 Wochen nach einer milden oder asymptomatisch verlaufenden Infektion mit *SARS-CoV‑2* auftritt. Seit Beginn der Erhebung bis KW 8/2022 wurden 735 Fälle im PIMS-Survey der DGPI gemeldet [11]. Die Prävalenz von *MIS-C/PIMS-TS* wird auf < 0,1 % der Infizierten geschätzt.

**Figure 1:**
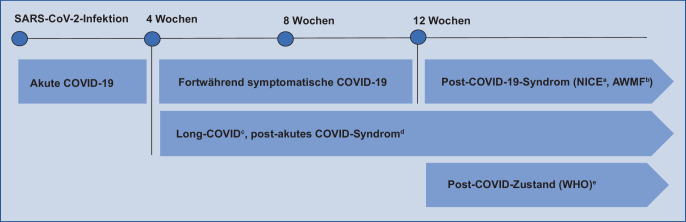

Bereits früh in der Pandemie berichteten vor allem Erwachsene über das Weiterbestehen, Wieder- oder Neuauftreten von Symptomen nach einer akuten *SARS-CoV‑2*-Infektion. Betroffene prägten den Begriff *Long COVID* und bezeichnen sich selbst als „*long haulers*“. Synonym mit *Long COVID* wurden für Symptome ab 4 Wochen auch die Begriffe *„post acute sequelae of COVID“* (PASC) [8] und *„post-acute COVID syndrome“* (PACS) [24] vorgeschlagen. Bis Herbst 2021 fehlte eine einheitliche Definition, die das Fortbestehen von Symptomen nach einer akuten COVID-19 charakterisiert. In der Leitlinie des *National Institute for Health and Care Excellence* (*NICE*) erfolgte eine Einteilung anhand der Dauer der Symptome nach akuter Erkrankung ([25]; Abb. 1). Dabei steht *Long COVID, „ongoing symptomatic COVID“* und *Post-COVID-Syndrom* für Symptome mit einer Dauer von mehr als 4 Wochen, von 4 bis 12 Wochen bzw. von mehr als 12 Wochen.

Einen Diagnosezusatzschlüssel für den „Zustand nach COVID-19“ hatte die Weltgesundheitsorganisation (WHO) Anfang 2021 mit ICD-10 U09.9! für den Fall vorgeschlagen, dass „der Zusammenhang eines aktuellen, anderenorts klassifizierten Zustandes mit einer vorausgegangenen COVID-19 kodiert werden soll“ [12]. Zusätzlich muss die führende Symptomatik verschlüsselt werden (z. B. „Atemnot“/R06, „Fatigue“/R52, „Kopfschmerzen“/R51 oder gegebenenfalls „myalgische Enzephalomyelitis/chronisches Fatigue-Syndrom (ME/CFS)“/G93.3).

Am 06.10.2021 definierte die WHO den „Post-COVID-19-Zustand“ (*„post-COVID-19 condition“*) für Erwachsene als eine Folgesymptomatik ab 3 Monaten nach sehr wahrscheinlicher oder nachgewiesener SARS-CoV-2-Infektion. Diese beinhaltet über mindestens 2 Monate persistierende, fluktuierende oder wiederkehrende für *Long COVID* typische Beschwerden (z. B. Fatigue, Kurzatmigkeit, kognitive Dysfunktion), die nicht durch eine andere Diagnose erklärt werden können und die Alltagsfunktion beeinträchtigen ([16], Abb. 1).

Für Kinder und Jugendliche wurde von der WHO noch keine endgültige Definition des „Post-COVID-Zustands“ vorgeschlagen. In Anlehnung an die o. g. Definition sollte der Zusatzcode U09.9! ab 3 Monate nach wahrscheinlicher oder gesicherter Infektion gewählt werden. 4 bis 12 Wochen nach COVID-19 sollten ein passender Code (U07 oder U08) sowie die führende Symptomatik verschlüsselt werden.

Im nachfolgenden Text sprechen wir zusammenfassend von *Long COVID*, also Beschwerden nach mindestens 4 Wochen, wenn nicht anders ausgewiesen.

Aufgrund der vielfältigen und zum Teil unspezifischen Art der Symptome sowie deren wechselhaftem Auftreten ist die Erfassung von *Long COVID* sehr komplex [36, 36]. Neben anderen Erkrankungen müssen Organschäden durch COVID-19 und deren Behandlung abgegrenzt werden. Das *„post-intensive care syndrome“* (PICS) tritt im Kindes- und Jugendalter eher selten auf [20]. Von besonderer Relevanz im Kindes- und Jugendalter ist die Differenzierung von Beschwerden, die als Folge der pandemiebedingten sozialen Veränderungen auftreten können („Long lockdown-Syndrom“) [29].

**Figure 2:**
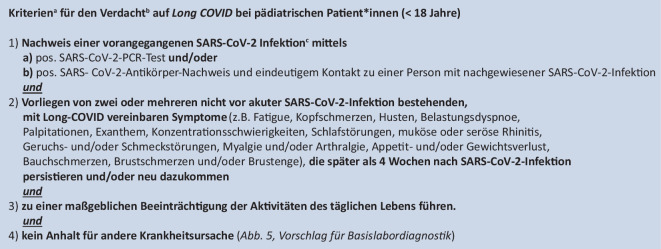

Wir empfehlen die Diagnose *Long* oder *Post-COVID* anhand der in Abb. 2 genannten Kriterien.

Im Erwachsenenalter schätzt die WHO die Prävalenz von postviralen Langzeitbeschwerden bei *SARS-CoV‑2*-Infizierten derzeit mit 10–20 % ein [16]. Mögliche Risikofaktoren sind ein initial schwerer Infektionsverlauf, bestimmte Grunderkrankungen und das weibliche Geschlecht. Zur Prävalenz im Kindes- und Jugendalter ist deutlich weniger bekannt [36]. Neben einigen Fallsammlungen wurden nur einzelne größere Kohorten- und Fall-Kontroll-Studien publiziert. Abhängig vom Studiendesign wird die Prävalenz von *Long COVID* bei Kindern und Jugendlichen in kontrollierten Studien mit 0,8–13 % angegeben [36]. In der CLoCK-Studie aus England wurde eine Prävalenz von 13 % drei Monate nach Infektion errechnet, allerdings basiert die Studie nur auf PCR-Tests ohne Serostatus, und der Rücklauf der Fragebogen war gering, sodass die Autoren eine Übererfassung von symptomatischen Patient*innen diskutieren [32]. Weitere Studien aus England sprechen eher für eine Häufigkeit von 4–5 % nach 4 Wochen und 1–2 % nach 8 Wochen [23], eine dänische Kohortenanalyse von 0,8 bei Schulkindern [4]. Bei SARS-CoV-2-positiven Jugendlichen traten im Vergleich zu einer Kontrollgruppe mehr Langzeitbeschwerden und Fehltage auf [17]. Auf ähnlich niedrige oder noch niedrigere Werte deuten auch seroepidemiologische Schuluntersuchungen aus Deutschland [3] und der Schweiz [28] hin.

Zum Schweregrad der Erkrankungen bei Kindern und Jugendlichen finden sich ebenfalls bislang wenige Daten. Insgesamt scheinen schwere Verläufe sehr selten zu sein [14]. Die Manifestation eines postviralen ME/CFS nach SARS-CoV-2-Infektion ist für das Adoleszenten- und Erwachsenenalter beschrieben [27]. Für Kinder liegen erst wenige Daten zu möglichen Risikofaktoren vor [35, 36].

**Figure 3:**
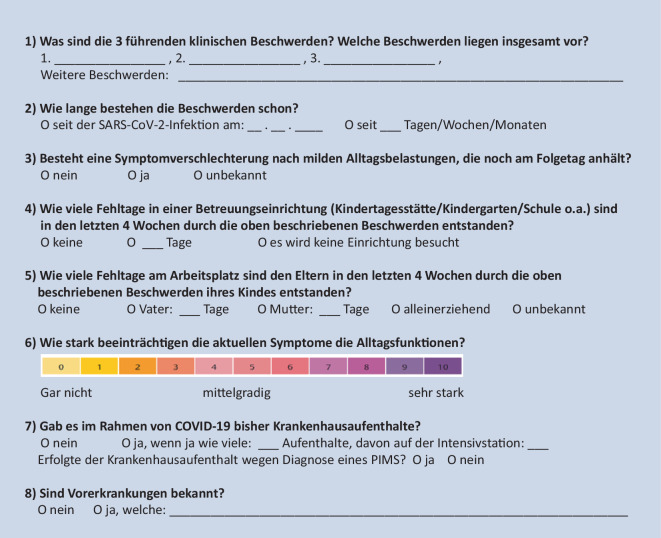

Zur Einschätzung der Dringlichkeit der ärztlichen Versorgung bei V. a. *Long COVID* wurden in einem Konsensusprozess der Arbeitsgruppe die in Abb. 3 genannten Fragen vorgeschlagen.

Eine erhöhte Dringlichkeit besteht, wenn mehr als 10 Fehltage in einer Betreuungseinrichtung per Monat den vergangenen Monaten und/oder eine sehr starke Beeinträchtigung des Alltags vorliegen.

Die Pathogenese von *Long COVID* ist bislang weitgehend ungeklärt. Verschiedene Erklärungsansätze weisen auf eine mögliche direkt durch das Virus vermittelte Organschädigung, Persistenz von Virusbestandteilen mit fortdauernder Immunaktivierung, Inflammation mit nachfolgender Fibrose und Autoantikörper-vermittelte Prozesse hin [5]. Untersuchungen der Retina zeigen eine gestörte Mikrozirkulation, die wahrscheinlich auf eine Endotheliitis mit endothelialer Dysfunktion zurückzuführen ist. Mikrozirkulationsstörungen könnten auch zu Funktionsstörungen anderer Organe, z. B. des Gehirns, beitragen und werden möglicherweise u. a. durch eine verringerte Verformbarkeit von Blutzellen hervorgerufen.

Zu therapeutischen Interventionen und zur Prognose sowohl von *Long COVID* als auch von ME/CFS nach *SARS-CoV‑2*-Infektion allgemein und insbesondere bei Kindern und Jugendlichen ist bislang wenig bekannt. Immunmodulatorische Ansätze und verschiedene Aphereseverfahren werden in Studien bislang vor allem bei Erwachsenen geprüft [5]. Umso wichtiger sind ein kontinuierlicher multizentrischer Erfahrungsaustausch unter Beteiligung aller pädiatrischen und internistischen Fachdisziplinen, die Patient*innen mit *Long COVID* betreuen, sowie eine gute Eigenerfassung der Beschwerden und Austausch Betroffener. Robustere Daten, v. a. durch gut konzipierte klinische Studien, sind notwendig, um präventive und therapeutische Konzepte zu etablieren [36].

### Visitenschema bei V. a. *Long COVID*

**Figure 4:**
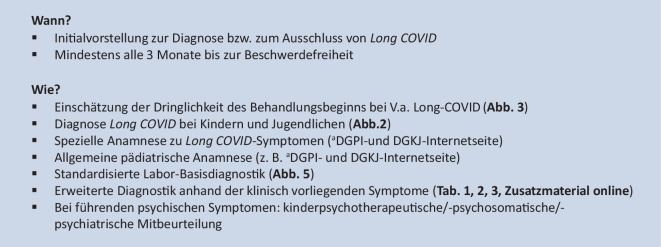

**Figure 5:**
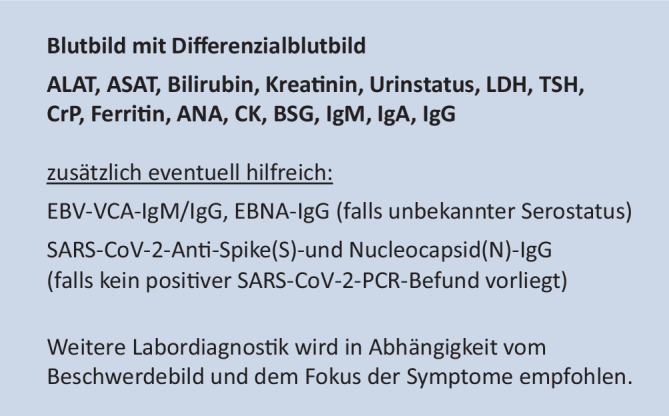

Zur Basisversorgung bei V. a. *Long COVID* wurden in einem Konsensusprozess Empfehlungen zum Visitenschema (Abb. 4) und zu Laboruntersuchungen (Abb. 5) erarbeitet. Zudem wurden konsentierte Fragebogen, darunter ein Erfassungsbogen zur zeit- und ressourcensparenden standardisierten Anamnese auf den DGKJ- und DGPI-Internetseiten hinterlegt. Empfehlungen zur erweiterten funktionellen und bildgebenden Diagnostik finden sich im **Zusatzmaterial online**.

In den nachfolgenden Kapiteln werden verschiedene, häufige mit *Long COVID* assoziierte Symptomenkomplexe behandelt. Eine Übersicht zur Häufigkeit einzelner Symptome bei Kindern und Jugendlichen findet sich bei Zimmermann et al. [36].

## Respiratorische und Herz-Kreislauf-Beschwerden

Respiratorische Beschwerden gehören zu den häufigsten Symptomen nach *SARS-CoV‑2*-Infektion bei Kindern und Jugendlichen. Bis zu 40 % geben nach 8 bis 12 Wochen noch persistierenden Husten, ca. 15 % noch Dyspnoe an [35]. Bei Kindern und Jugendlichen werden persistierende Beschwerden nur in ca. 5 % beschrieben [4]. Eine bronchiale Hyperreagibilität ist, wie nach anderen viralen Atemwegsinfektionen, möglich und kann sich durch Inhalation mit Kortikosteroiden bessern. Als Ursache für die subjektiv empfundene Dyspnoe ist eine autonome Dysregulation denkbar. Auch Trainingseffekte und funktionelle Atemstörungen können eine Rolle spielen [18]. Letztere können durch spezielle Atemtherapie behandelt werden. Selten sind Ventilation-Perfusion-Inhomogenitäten detektierbar.

Kardiale Beschwerden wie Palpitationen oder auch Thoraxschmerzen werden von 2–31 % der Kinder und Jugendlichen 8 bis 12 Wochen nach *SARS-CoV‑2*-Infektion berichtet [35]. In den meisten Fällen von *Long COVID* lässt sich kein organisches Korrelat für die Beschwerden finden, weder in der Bildgebung noch bei den Laborparametern. Ausgeschlossen werden sollte eine Myokarditis, welche bei 0,045 % der infizierten Jungen bis zu 82 Tage nach der Infektion auftrat. Mädchen sind seltener betroffen gewesen. Diagnostik und Behandlung sind identisch zu anderen Virusmyokarditiden. Bei PIMS ist das kardiovaskuläre System bei > 50 % der Erkrankten beteiligt, und die meisten Residualbefunde betreffen den Kreislauf (Koronaraneurysmen). Für die Nachsorge nach PIMS gibt es eine eigene Empfehlung. Nach Impfungen mit mRNA-Impfstoffen traten ebenfalls gehäuft Myokarditisfälle auf. Auch wenn diese milder verlaufen als nach Infektion, sollte derselbe Algorithmus angewendet werden. Am häufigsten betroffen sind männliche Jugendliche nach der Zweitimpfung (1:17.000); bei Mädchen ist das statistische Risiko nicht relevant erhöht. Rhythmusstörungen können bei jeder Form der kardialen Beteiligung auftreten, sowohl bradykarde (AV-Block) als auch tachykarde (ventrikuläre Tachykardien). Die Behandlungen dieser Erkrankungen sollte durch Kinderkardiolog*innen entsprechend den Leitlinien der DGPK erfolgen. Davon abzugrenzen sind Sinustachykardien, die oftmals Ausdruck der vegetativen Dysregulation sind. Bis zu 30 % der Betroffenen zeigen auch eine orthostatische Dysregulation, z. B. im Sinne eines posturalen (orthostatischen) Tachykardiesyndroms (POTS) [33]. Letzteres wird durch einen angelehnten 10-Minuten-Stehtest oder eine Kipptischuntersuchung diagnostiziert [19]. Dabei kommt es durch Aufrichten zu Symptomen der orthostatischen Intoleranz und einem Anstieg der Herzfrequenz bei stabilem Blutdruck. Der Leidensdruck der Betroffenen kann erheblich sein und den Schulunterricht in aufrechter Position beeinträchtigen. Die Behandlung zielt darauf ab, das intravasale Volumen zu erhöhen (salzreiche Kost, erhöhte Flüssigkeitszufuhr), das Blutvolumen umzuverteilen (Kompressionsstrümpfe, Körperposition), auslösende oder verschlechternde Faktoren zu vermeiden (z. B. große Mahlzeiten) und, soweit möglich, eine körperliche Aktivierung zu erreichen. Eine medikamentöse Behandlung kann in ausgeprägten Fällen in Rücksprache mit einem spezialisierten Zentrum versucht werden (Fludrocortison, Midodrin, Ivabradin, β‑Blocker). Physio- und ergotherapeutische Konzepte können die Symptome mildern, müssen aber an die Belastbarkeit der Betroffenen angepasst werden.

## Myalgien, Myositis, Arthralgien, COVID-Zehen

Spezifische rheumatologische Symptome sind bei *Long COVID* selten. Hingegen werden Myalgien und Arthralgien je nach Studie von 1–61 % der Kinder und Jugendlichen mit *Long COVID* berichtet [35, 36]. Eine Myositis mit erhöhten Spiegeln der Kreatininkinase ist möglich. Gliederschmerzen sind eines der klinischen Diagnosekriterien von ME/CFS [10, 13].

Bei *„Chilblain“*-ähnlichen Läsionen (*„chilblain-like lesions“*, CLL) handelt es sich um schmerzhafte Entzündungsreaktionen kleiner Blutgefäße der Haut mit Minderdurchblutung. Klinisch imponieren rötliche bzw. bläulich-livide, akrale Schwellungen, in erster Linie der Zehen (*„COVID toe“*) und Finger. Klinisch sind die Läsionen nicht von Frostbeulen (*Perniones*) oder dem *Chilblain Lupus*, einer kutanen Manifestation des systemischen Lupus erythematodes (SLE), zu unterscheiden. Kälte ist kein zwingender Auslöser. Juckreiz ist möglich [7]. Die Prävalenz von *COVID*-Zehen im Kindesalter ist nicht bekannt. 10 % der Betroffenen berichteten über ein familiäres Auftreten [7]. Der Verlauf bei *COVID*-Zehen ist meist mild und eine Therapie nicht erforderlich; bei moderatem oder schwerem Verlauf wird wie bei *Perniones* oder *Chilblain Lupus* behandelt. Die mögliche Wirkung von nichtsteroidalen Antiphlogistika (NSAID), Antihistaminika, topischen oder systemischen Steroiden sowie Kalziumantagonisten ist bislang in Studien nicht belegt. Schwere Verläufe mit Zeichen der Ischämie erfordern eine rasche rheumatologisch-hämostaseologische Diagnostik (s. DGKJ/DGPI-Internetseiten), eventuell eine kombinierte immunsuppressive und vasodilatative Therapie sowie Antikoagulation.

## Riech- und Schmeckstörung

Die Prävalenzangaben zu Riechstörungen in pädiatrischen Studienpopulationen schwanken zwischen 5 und 40 %. Höhere Prävalenzen werden bei standardisierter Riechtestung im Vergleich zur Selbsteinschätzung der Riechfunktion ermittelt [6]. Dies unterstreicht die Bedeutung standardisierter Riechtests, z. B. des „U-Sniff“-Geruchsidentifikationstests oder des „Smell Wheel“ [30]. COVID-assoziierte Schmeckstörungen sind wesentlich seltener als COVID-assoziierte Riechstörungen [9]. Die Prognose letzterer wird als günstig angesehen. In den meisten Fällen kommt es nach wenigen Tagen bis Wochen zu einer spontanen Besserung. Es sind jedoch auch Riechstörungen länger als 120 Tage beschrieben. Im Rahmen der Erholung können Geruchsveränderungen auftreten. Sie sind temporär und verschwinden mit der Erholung typischerweise nach 6 bis 12 Monaten. Sollte eine Riechstörung länger anhalten, kann ein Therapieversuch mittels Riechtraining (konsequente, repetitive, kurze Geruchsexpositionen) durchgeführt werden. Eine neu aufgetretene, nicht anders erklärte Riechstörung (ohne eindeutige andere Ursache) sollte in der aktuellen Pandemie an eine *SARS-CoV‑2*-Infektion denken lassen. Neue Daten aus der Omikron-Welle legen nahe, dass durch die neue Virusvariante weniger Geschmacksverlust induziert wird.

## Fatigue, post-exertionelle Malaise und Schlafstörungen

Eines der häufigsten Symptome bei *Long COVID* ist altersübergreifend die postvirale Erschöpfung (Fatigue). Fatigue ist nicht mit Müdigkeit gleichzusetzen. Die krankhafte Erschöpfung resultiert nicht selten in einer deutlich eingeschränkten Alltagsfunktion sowie reduzierter Lebensqualität und Teilhabe. Im Kindes- und Jugendalter lag die Prävalenz studienabhängig bei 3–87 % der Infizierten [35, 36].

Bei manchen Betroffenen verschlechtern sich die Symptome von *Long COVID* nach geringer oder moderater Alltagsbelastung über mehrere Stunden, tage- oder wochenlang. Dieses Phänomen wird post-exertionelle Malaise (PEM) genannt. Eine PEM an Folgetagen der Belastung ist das Leitsymptom von ME/CFS. ME/CFS muss abgeklärt werden, wenn neben Fatigue und PEM auch Schmerzen, Schlafstörungen, autonome, neurokognitive, neuroendokrine und/oder immunologische Störungen vorliegen [27]. Die Diagnose ME/CFS wird nach angemessen breiter Differenzialdiagnostik und anhand bestimmter klinischer Kriterien gestellt [10, 13]. Fragebogen zu Fatigue und PEM sowie ein pädiatrisches ME/CFS-Arbeitsblatt (MBSQ) werden auf den DGKJ- und DGPI-Internetseiten bereitgestellt.

Bei ausgeprägter PEM ist eine Anleitung zu konsequentem *Pacing *(Schritthalten mit den eigenen Energiereserven) erforderlich: Der Alltag soll so gestaltet werden, dass Überlastungen mit PEM vermieden und Alltagsaktivitäten bestmöglich aufrechterhalten werden. Ein Aktivitäts- und Symptomtagebuch kann die Einschätzung der individuellen Belastungsgrenzen erleichtern. Alle Behandlungsmaßnahmen müssen an Letztere angepasst werden [25]. Entspannungstechniken können hilfreich sein. Eine starre stufenweise Aktivierung ist bei ME/CFS kontraindiziert, da sie zu schwerer PEM führen kann. Wenn eine Fatigue ohne PEM vorliegt, kann eine therapeutische Aktivierung versucht werden.

Schlafstörungen können postviral isoliert oder mit anderen Symptomen kombiniert auftreten [22] und ursächlich für Symptome am Tag sein. Sie treten im Kindes- und Jugendalter generell mit einer Häufigkeit von 25–40 % auf und bedürfen bei erhöhtem Leidensdruck und/oder Persistenz einer differenzialdiagnostischen Einordnung und ggf. Therapie [34].

## Neurologische und psychiatrische Symptome, Entwicklungsstörungen

*Long COVID* sollte bestmöglich von Langzeitfolgen der pandemiebedingten Belastungen abgegrenzt werden. Da Komorbiditäten vorliegen können, ist es wichtig, somatische und psychiatrische Befunde in der Gesamtschau zu bewerten.

Kritische physische Leistungsminderung (evtl. mit PEM), klinisch reproduzierbare fokale oder generalisierte Muskelschwäche, Bewegungsstörungen sowie testpsychologisch nachweisbare kognitive Einschränkungen (insbes. Aufmerksamkeit, Gedächtnis) sind richtungweisend für *Long COVID* [8]. Bei Vorliegen von Symptomen, die auch bei anderen neuropädiatrischen Erkrankungen häufig sind (z. B. Schmerzen, Müdigkeit, Schlafstörungen, Parästhesien), ist eine erweiterte Differenzialdiagnostik, auch unter Einbezug neurophysiologischer, bildgebender und testdiagnostischer Verfahren, angezeigt.

Bei der Beurteilung neurokognitiver und/oder psychischer Störungen sollten das prämorbide Niveau berücksichtigt und pandemiebedingte, reaktive oder genuine psychische Störungen abgegrenzt werden [29, 31]. Erste Hinweise geben subjektive Angaben von Kindern, Eltern oder anderen Bezugspersonen in Fragebogen (s. DGKJ/DGPI-Internetseiten). Eine ausführliche neuropsychologische Untersuchung ist bei neu aufgetretenen Auffälligkeiten in einem der kognitiven und/oder sozial-emotionalen Bereiche bzw. Schulleistungsproblemen empfohlen. Zusätzliche ophthalmologische und phoniatrische Untersuchungen können hilfreich sein. Vor allem bei unspezifischen, häufigen Symptomen (z. B. Kopfschmerzen, Konzentrationsstörungen) sind eine Differenzierung und Verlaufsbeurteilung von zentraler Bedeutung. Depressive und ängstliche Symptome haben bei Kindern und Jugendlichen im Zuge der Pandemie zugenommen [29]. Die Differenzialdiagnostik ist nicht selten schwierig. Wichtige Differenzialdiagnosen sind somatoforme Störungen, Anpassungsstörung und Aufmerksamkeitsdefizit‑/Hyperaktivitätsstörung (ADHS). Aus diesem Grunde ist eine kinder- und jugendpsychiatrische oder kinder- und jugendpsychotherapeutische Differenzialdiagnostik immer notwendig, wenn postviral prominente psychische Symptome auftreten.

Die neuropädiatrische Behandlung erfolgt symptombezogen unter Berücksichtigung des individuellen physischen und kognitiven Leistungsniveaus. In der Anfangsphase der Behandlung ist eine engmaschigere Verlaufsbeobachtung notwendig, um das Therapiekonzept anzupassen („shaping“) und so eine PEM zu verhindern. Für schwere und mittelschwere Krankheitsverläufe mit erheblicher Beeinträchtigung der Alltagsteilhabe (Haushalt, Kita, Schule, Freizeitaktivitäten) sollte eine stationäre, neurologische Rehabilitation mit spezifischen Behandlungskonzepten angeboten werden. Bei Kindern mit neurokognitiven Symptomen wird ein entwicklungsneuropsychologisches Monitoring im Langzeitverlauf über mehrere Jahre empfohlen [31], da Folgen zerebraler Funktionsstörungen oftmals erst viele Jahre später erkennbar sind [21]. Entwicklungs- sowie schulische Lernstörungen resultieren zudem nicht selten in sekundären sozialen und emotionalen Störungen.

Bei Vorliegen von primären oder sekundären psychischen Störungen (z. B. Depression, Angst‑/Zwangs‑/Traumafolgestörungen) erfolgt nach zwingend notwendiger kinder- und jugendpsychiatrischer oder approbierter kinder- und jugendpsychotherapeutischer Indikation eine leitliniengerechte Intervention, die ggf. somatopsychische Wechselwirkungen interdisziplinär angemessen berücksichtigt. Im Gegensatz zu ME/CFS (*Pacing*) muss einer depressiven Episode durch stressarme Aktivierung begegnet werden. Die Interventionen bei neuropsychologischen Auffälligkeiten sollten in Kooperation mit der Schule erfolgen (z. B. Nachteilsausgleich, sonderpädagogische Förderung). Kindzentrierte Interventionen (neuropsychologische Therapie, ggf. Ergotherapie) können zur Restitution bzw. zur Kompensation von Teilfunktionen eingesetzt werden.

## Diagnostik bei Verdacht auf *Long COVID*

Anhand der anamnestisch und klinisch ermittelten Hauptsymptome wird ein gestuftes, diagnostisches Vorgehen empfohlen, welches in Abb. 4 dargestellt ist. Dieses beinhaltet eine Basis-Labordiagnostik (Abb. 5). Zusätzlich kann die Erfassung von patient*innenberichteten Ergebnissen (*„patient-reported outcome measures“,* PROM) hilfreich sein. Vorschläge für diesbezügliche Erfassungsbogen sind auf den Internetseiten der DGKJ und DGPI hinterlegt. Die Fragebogen sind *per se* nicht geeignet, um eine Diagnose zu bestätigen oder zu verwerfen, sondern können nur Anhaltspunkte liefern für Differenzialdiagnosen und eine Überweisung in eine spezialisierte Einrichtung.

Zur weiteren Einschätzung der Symptome und des Verlaufes kann in Absprache mit den Familien ein Heimmonitoring der Symptome in einem Symptomtagebuch, aber auch je nach Symptomatik durch Monitoring von Vitalparametern und Belastbarkeit (z. B. einminütiger *Sit-to-stand*-Test) in enger Absprache mit den Behandlern sinnvoll sein.

## Supplementary Information




