# Supplementary material for: Recommendation for standardized medical care for children and adolescents with long COVID: Statement of a multidisciplinary pediatric expert group of the DGKJ convent societies (status: February 2022)
Source: Monatsschr Kinderheilkd. 2022 May 25;170(6):539–47. [Article in German] doi: 10.1007/s00112-021-01408-1 (PMC9131710; doi:10.1007/s00112-021-01408-1)
Supplement: Supplementary file 1 [file 112_2021_1408_MOESM1_ESM.pdf]

Online Supplement:

Tabelle 1: Erweiterte Labordiagnostik

**Erweiterte Labordiagnostik:**	**Erstvorstellung:**<br>Frühestens 4-6 Wochen nach SARS-CoV-2-Infektion	**Folgetermine**<br>3, 6, 9, 12, 18, 24 Monate, falls noch symptomatisch
Bei gezielter ***kardiologischer Fragestellung***	*u.a. CK, Troponin I/T, NT-proBNP, D- Dimere (siehe PIMS)*	Nach klinischer Indikation und Vorbefunden
Bei gezielter ***gastroenterologischer Fragestellung***	*u.a. ASAT (GOT), GGT, Harnstoff, Gesamteiweiß, Albumin, Lipase, Alkalische Phosphatase, ggf. Calprotectin im Stuhl, Blut im Stuhl, ggf. anti-Transglutaminase oder anti-Endomysium IgA mit Bestimmung von Gesamt IgA*	Nach klinischer Indikation und Vorbefunden
Bei erhöhtem Risiko für ***thrombotische Ereignisse u./o. Antikoagulation***	*u. a. INR, PTT, D-Dimere, Fibrinogen, Protein S und C, Antiphospholipid Ak, Konsil Hämostaseologie*	Nach klinischer Indikation und Vorbefunden
Bei ***Fatigue, PEM u./o. neurologischen Symptomen***	*u.a.* venöse BGA, Laktat, Glukose Natrium, Kalium, Chlorid, Calcium, Phosphat, Magnesium, ACTH, Cortisol basal, TSH, fT4, ggf. Vitamin B12, B6, B1, Folsäure, Zink, Vitamin D3, Ausschluss Sjögren (IgG, anti-Ro, anti-LA, ANA, IgM Rheumafaktor) oder andere rheumatologische Erkrankung (s.u.)<br>Liquorstatus	Nach klinischer Indikation und Vorbefunden
Bei ***Hautveränderungen und gezielter rheumatologischer Fragestellung bzw. V.a. Vaskulitis***	*u.a.* ENAs, ANCA, Antiphospholipid-Antikörper, Lupus-Antikoagulans (siehe PIMS), U1-RNP-Antikörper, anti-Ro AK anti-LA, ggf. auch weitere Autoantikörper durch Rheumatologie	Nach klinischer Indikation und Vorbefunden

Online Supplement:

Tabelle 2: Bildgebende Diagnostik

**Bildgebende Diagnostik**	**Erstvorstellung**: Frühestens 4-6 Wochen nach SARS-CoV-2-Infektion	**Folgetermine** 3, 6, 9, 12, 18, 24 Monate, falls noch symptomatisch
**Bei allen Patient*innen**	Nach klinischer Indikation	Nach klinischer Indikation
Bei gezielter ***rheumatologischer oder infektiologischer Fragestellung***	EKG, wenn auffällig Echokardiographie, Abdomen-Sonogramm, Röntgen-Thorax, ggf. Gelenksonografie	Nach klinischer Indikation
Bei gezielter ***kardiologischer Fragestellung***	EKG, Echokardiographie, ggf. Kardio- MRT	EKG, Echokardiographie
Bei gezielter ***pulmonologischer Fragestellung***	Röntgen-Thorax, Lungen-Sonogramm, ggf. *low dose* CT- Thorax ohne Kontrastmittel (*z. A. pulmonale Infiltrate, Belüftungsstörungen*)	Nach klinischer Indikation und Vorbefunden: Lungen-Sonogramm/ Röntgen-Thorax, ggf. *low dose* CT-Thorax, MRT-Lunge
Bei gezielter ***gastroenterologischer Fragestellung***	Abdomen-Sonogramm (Fokussuche: Raumforderung/ Entzündlicher Prozess)	Abdomen-Sonogramm, ggf. Gastro-/Koloskopie
Bei gezielter ***neurologischer Fragestellung*** **(inkl. HNO/Ophthalmologie)**	MRT Schädel/spinal bei neuropädiatrischer Indikation (z.B. fokale neurologische Symptome, bzw. Verschlechterung oder Neuhinzutreten neurologischer Symptome)	Nach klinischer Indikation

Online Supplement:

Tabelle 3: Funktionsdiagnostik

**Funktionsdiagnostik**	**Erstvorstellung**<br>Frühestens 4-6 Wochen nach SARS-CoV-2-Infektion	**Folgetermine**<br>3, 6, 9, 12, 18, 24 Monate, falls noch symptomatisch
Bei allen Patient*innen	Standard: Blutdruck, SpO2, EKG, Orthostase-Test (10-min-Stehtest, Kipptisch) z.A. autonome Dysregulation, Spirometrie und SpO2 nach freiem Laufen, Belastungstest (6 min Gehtest, 1 min *Sit-to-stand*-Test), Neurostatus (SOP im Anhang), Screening kognitiver Funktionen und psychischer Probleme (KOPKI bzw. KOPKIJ und CBCL/YSR)	Nach klinischer Indikation organspezifische Abklärung und ggf. neuropsychologische Diagnostik
Zusätzlich bei kardialer Symptomatik	Ruhe-12-Kanal-EKG und Rhythmusstreifen, ggfs 24h-Langzeit-EKG/-RR, Belastungs-EKG, Spezialdiagnostik: Verfahren zur Beurteilung von Herzfrequenzvariabilität und endothelialer Dysfunktion	Nach klinischer Indikation, insbesondere: 24h-Langzeit-EKG, Belastungs-EKG
Zusätzlich bei ***pulmonaler Symptomatik***	s.o., Bodyplethysmographie, wenn verfügbar Laufbandbelastung mit Broncholyse und BGA	Nach klinischer Indikation, insbesondere: CO-Diffusion, LCI, Spiroergometrie mit BGA
Zusätzlich bei ***neurologischer Symptomatik*** *(inkl. HNO/Ophthalmologie)*	s.o., EEG, ggf. standardisierter Riechtest (z.B. U-Sniff), ggf. quantitative sensorische Testung (QST, z.B. als *bedside*-Test)	Nach klinischer Indikation, insbesondere: NLG bei peripherer neurol. Störung, Testung Schwindel (HNO), Visusprüfung (Augenarzt)
Zusätzlich bei ***Fatigue**** **/ Post-exertioneller Malaise (*PEM*)**	MBSQ, PedsQL MFS, DSQ-PEM-Score, PHQ4, Ausschluss psych. Differentialdiagnosen (wie Depression, Angststörung)	Nach klinischer Indikation, insbesondere Wiederholung PedsQL MFS, DSQ-PEM-Score, Erweiterung nach klinischer Indikation, ggf. psychologische Mitbetreuung
Zusätzlich bei ***Lernproblemen und/oder psychischen Auffälligkeiten***	Neuropsychologische Diagnostik	Monitoring der Lern- und psychischen Störungen
